# A degree-day model for forecasting adult phenology of *Popillia japonica* (Coleoptera: Scarabaeidae) in a temperate climate

**DOI:** 10.3389/finsc.2022.1075807

**Published:** 2022-12-15

**Authors:** Dominique N. Ebbenga, A. A. Hanson, E. C. Burkness, W. D. Hutchison

**Affiliations:** ^1^ Department of Entomology, University of Minnesota, St. Paul, MN, United States; ^2^ University of Minnesota Extension, Extension Integrated Pest Management (IPM) Program, Morris, MN, United States

**Keywords:** Japanese beetle, *Popillia japonica*, pest phenology, modeling, invasive species

## Abstract

Japanese beetle, *Popillia japonica* (Newman), was first detected in the United States in New Jersey in 1916. The beetle gradually spread to the Midwest U.S. region, and was first confirmed in Minnesota in the late 1960’s. *Popillia japonica* has subsequently become a major invasive insect pest in turfgrass and several agricultural crops. As *P. japonica* continues to spread throughout the U.S., and other countries, it is important to develop efficient ways to monitor adult populations, and where possible, forecast the phenology of adult population dynamics. During 2019-2021, field trials were conducted to develop a degree-day model that can be used to forecast *P. japonica* adult phenology under Minnesota, and Midwest summer climatic conditions in. We used commercially available traps and lures to monitor adult flight phenology, specifically beetle trap-catch, along with weather data at four locations in Minnesota, to relate ambient field temperatures to population phenology. The concordance correlation coefficient (*CCC*), an index of both precision and accuracy, was used to develop a final degree-day model. Model development included evaluation of simple and sine-wave degree-day calculation methods, start dates between 1 Jan. and 1 April, and a range of lower (0-15 °C) and upper (20-37 °C) thresholds. The optimum model was found to be a simple degree-day calculation, using a biofix date of 1 Jan, and lower and upper thresholds of 15 and 21.7 °C, respectively, for predicting 10% beetle trap-catch. The model will aid in future integrated pest management (IPM) and regulatory strategies by providing a tool for prediction of *P. japonica* adult flight phenology.

## Introduction

Japanese beetle, *Popillia japonica* Newman (Coleoptera: Scarabaeidae), is an invasive species first detected in 1916 in New Jersey, following an accidental introduction from Japan, on imported rootstock (1). *Popillia japonica* was first detected in Minnesota in 1968, gradually increased in abundance (2), and has only recently become a dominant pest, since 2010 (3, 4). The beetle is currently a major invasive insect pest of turf, residential ornamentals, and several agricultural crops in the Midwest U.S. region (5–7).

*Popillia japonica* typically exhibits a univoltine life cycle in the Midwest (8), where immature stages reside in the soil and overwinter as late instar larvae (9, 10). Once *P. japonica* has completed pupation in spring, adults eclose and emerge from the soil to seek mates, and host plants for nutrition; adult longevity ranges from 4-6 weeks (9). Although several trapping and behavior studies have been conducted to understand adult biology (6, 11), most of the research to date has been directed toward understanding the development of the immature stages and the larval damage inflicted upon turfgrass (11, 12). In recent years, however, concerns have been raised regarding the biology and impact of the adult beetles, particularly for several horticultural and agricultural crops in the Midwest region (5, 6). With over 300 different host plants, there are many opportunities, even in northern tier, temperate climates such as Minnesota, for *P. japonica* adults to cause substantial defoliation, mainly consuming leaf tissue within plant canopies (7, 9, 11). Adult feeding can be very concerning to producers as they observe heavy defoliation with little knowledge or predictability as to how the infestation may impact their crops.

Currently, *P. japonica* has spread to at least 36 states in the U.S. (6, 13). Since 2014, concerns of *P. japonica* invading new regions have caused some states, with no known established populations, to implement quarantine protocols for either early detection or to assist in mass trapping activities to help prevent an introduction of *P. japonica* (13). However, in areas where *P. japonica* has established, monitoring the pest has become important for determining the geographic extent of invaded areas, or used to inform producers of the potential threats to high-value crops. Commercially available traps are currently used for monitoring *P. japonica* (14–17) and the use of an in-field, volumetric approach for rapid processing of trap samples was recently validated (17). However, traps used for monitoring only give notification of pest activity following emergence or dispersal to specific field sites. Beyond weekly trapping, another way to utilize trap-catch data is the development of models for the purpose of forecasting emergence, or insect phenology throughout the season (18–20).

Degree-day models are a useful tool for growers, crop consultants and researchers to forecast or predict the phenology of various important insect life-stage events (e.g., 19, 21), such as first emergence or peak (50%) adult emergence. Clearly, access to local or regional ambient temperature data is also critical for accurate degree-day modeling and forecasts. For the Midwest climate, *P. japonica* adults typically becomes active from mid-June to early July and continue to feed on several crops through early September (4). However, as climates in the Midwest continue to moderate with milder winters, and warmer springs (22), this general timeframe of insect activity can differ greatly. Currently for *P. japonica*, there are no established degree-day models for adult activity in the Midwest region.

Most recently, research on *P. japonica* has increasingly focused on adult beetle feeding injury and the need for improved integrated pest management (IPM) strategies for several fruit and agricultural crops (5–7, 23). Given the extent of *P. japonica* feeding injury, it is critical to develop new tools that can be shared with growers, crop consultants, and researchers to monitor *P. japonica* before feeding damage occurs. Therefore, research was conducted during the summers of 2019–2021 to develop a model that can be used to better track and forecast *P. japonica* adult phenology throughout the growing season, in a temperate, Midwest U.S. climate. In this study, we used commercially available traps and lures to monitor adult *P. japonica* activity at four locations in southern Minnesota, to better define adult *P. japonica* population dynamics throughout the year. Specifically, our objective was to develop a degree-day model to improve current IPM strategies and regulatory planning for adult *P. japonica* monitoring.

## Materials and methods

### Monitoring adult *P. japonica*

During 2019- 2021, *P. japonica* Trécé™ traps (Trécé™, Adair, OK) were deployed near raspberry crops at both the Rosemount Research and Outreach Center, near Rosemount, MN (RROC), MN (44° 43’ N, 93° 05’ W), and Forest Lake, MN (45° 13’ N, 92° 53’ W). Two additional trapping sites in vineyards were located near Hastings (44° 41’ N, 92° 52’ W) and the Horticultural Research Center (HRC), University of Minnesota, in Excelsior (44°52’ N, 93°38’ W), MN. Soils at each location were classified as follows: RROC had approximately 0.1% sand, 62.4% silt, and 37.6% clay; Forest Lake had 58.8% sand, 15% silt, and 26.2% clay; Hastings had 71.2% sand, 5% silt, and 23.8% clay; HRC had 32.5% sand, 33.8% silt, and 33.8% clay. Trécé traps, paired with semiochemical based lures containing a blend of the *P. japonica* sex pheromone and floral compounds (7, 14–16), were used for all traps. The lure used in traps is the commercially available *P. japonica* dual lure system. The dual lure consists of a food bait (phenethyl propionate + eugenol + geraniol (3:7:3)) and the synthetic sex pheromone known as ‘Japonilure’ ((R,Z)-5-1-decenyl)dihydro-2(3H)-furanone) (16). Since the lure has both a food and pheromone component, it is highly effective in attracting and capturing both male and female *P. japonica*, making for an ideal lure to monitor populations (7, 14, 15).

Samples were collected twice per week in 2019 and 2021; however, due to COVID-19 restrictions in 2020, traps were collected once per week. Trap samples were processed in the field using a volumetric measurement method established by Ebbenga et al. (7). Trap contents were placed in an Accu-pour™ measuring pitcher (Gemplers, Janesville, WI), with a capacity range of 100 – 2000 ml, rounded to the nearest 100 ml. For beetle samples that were <100 ml, a smaller Accu-pour beaker (Gemplers, Janeville, WI) was used, consisting of a range from 20 – 500 ml. In 2019, three traps were placed at each location on 3 Jun., and in 2020 and 2021, trap number was increased to 4 at each location and traps were deployed on 9 Jun. and 25 May, respectively, well before the first beetles were captured. Traps were secured to green metal stakes approximately 1 m above the soil surface, and were set approximately 10 m apart. Due to constraints with research locations and allowability of trap deployment, greater distances between traps were not achievable.

### Temperature data

Each year, minimum and maximum daily temperatures were collected from local weather stations and HOBO temperature loggers (Onset Computer Corporation, Bourne, MA) depending on the location. Locations in Forest Lake and Hastings used weather station data collected from the Minnesota State Climatology office (https://climateapps.dnr.state.mn.us/index.htm) operated and maintained by the Department of Natural Resources, Division of Ecological and Water Resources. For these locations, all weather stations were within 16 km of trap locations. For the HRC, an onsite weather station was used, and data were collected from the NEWA website operated and maintained by Cornell (newa.cornell.edu), which is the preferred weather station used on this research site. Finally, temperatures collected from RROC were collected using an Onset HOBO MX2303 wireless temperature data logger (Onset Computer Corporation, Bourne, MA). The Onset HOBO MX2303 was set to read ambient air temperature every 2 hours 7 days per week. The temperature probe recording ambient air temperatures was secured to a green metal stake adjacent to a raspberry patch and sheltered from direct sunlight. Temperature data collected at RROC was compared to nearby local weather stations to confirm daily minimums and maximums were similar, prior to use in model development.

### Model development and validation

Model development and validation was based on the approach developed by Hanson et al. (19), where field-based trap-catch pest data are used to seek optimal model solutions. This was done by using multiple locations to reflect natural variability in pest phenology, and for multiple years to reflect variable weather scenarios. Model development was conducted using multiple site-year data sets, by iterating through all possible combinations of model start dates, lower and upper thresholds, and calculation methods for degree-days (19). Separate, independent data sets were selected for several site-years for model validation. All calculations and analyses were performed according to Hanson et al. (19), using R version 4.1.2 (24).

To partition model development and validation data, the 12 site-years were divided in half and randomly selected for model development (n=6), while the remaining data sets were used for model validation (n=6) (**Table 1**). For model building, four start dates often used in the Midwest U.S., were included, Jan. 1, Feb. 1, March 1, and April 1, and converted to Julian dates of 1, 32, 50, and 91, respectively (19). Lower and upper threshold parameters, respectively, ranged from 0-15 °C (32-59°F) and 20- 37 °C (68-98.6°F), respectively, increasing by 0.56°C (1°F) increments. Degree-day calculations were performed using both a simple average degree-day method (25, 26) and the half-day sine-wave method (27), for a total of 7,392 degree-day model parameter combinations. Simple degree-days were calculated according to McMaster and Wilhelm (28) using the average of observed daily maximum and minimum temperatures minus the lower developmental threshold (i.e., method 1), which is not to be confused with another common simple-degree method (i.e., method 2) that, before averaging, changes the observed daily maximum or minimum temperature to equal to the lower developmental threshold if either falls below that threshold.

**Table 1:** Randomized location datasets used for *P. japonica* model development and validation, Minnesota, 2019-2021.

Location	Development dataset year	Validation dataset year
Rosemount	2020	2019, 2021
Hastings	2020	2019, 2021
Forest Lake	2019, 2021	2020
Excelsior	2019, 2021	2020

For each combination of start date, upper and lower threshold, and calculation method, logistic regression [eq. 1] was performed using the six-model development site-years


eq. 1 $$prop.emergence=\frac{e^{\ln(D)s+i}}{1+e^{\ln(D)s+i}}$$

where *D* = degree-days, *s* = slope, and *i* = intercept. Degree-days were natural-log transformed to use a log-logistic distribution for improved model fit with accumulated annual degree-days as an independent variable and proportion cumulative percent adult emergence as the dependent variable. Observed degree-days for 10% trap-catch were determined from each regression model to generate predicted dates of 10% trap-catch activity in the model development data.

To assess the performance of the models, *via* predicted versus observed dates of 10% trap-catch, the concordance correlation coefficient (*CCC*) was used (19). The *CCC* was selected because it reflects both precision (*r*) and accuracy (*A*) where *CCC*= *rA.* (29–31). Typical *CCC* values range between 0 and 1 with 1 being a perfectly precise and accurate model, and 0 representing no precision or accuracy (19, 32).

During development, the model with the highest *CCC* value was selected and used for model validation. Model validation used the target date of 10% trap-catch to determine the best performing model, given observed trap-catch and utilized data from the remaining 6 site-years (**Table 1**). While *CCC* was primarily used to rank models for 10% trap-catch, the Akaike information criterion (*AIC*) was also used to compare fit across the entire logistic distribution. When comparing *AIC* values across models, the lowest value indicates for best agreement across the distribution, and these values are on an unbounded scale, so *AIC* is only used for relative comparisons between models rather than absolute measures of fit (33).

## Results

### Monitoring adult *P. japonica*

**Figure 1:**
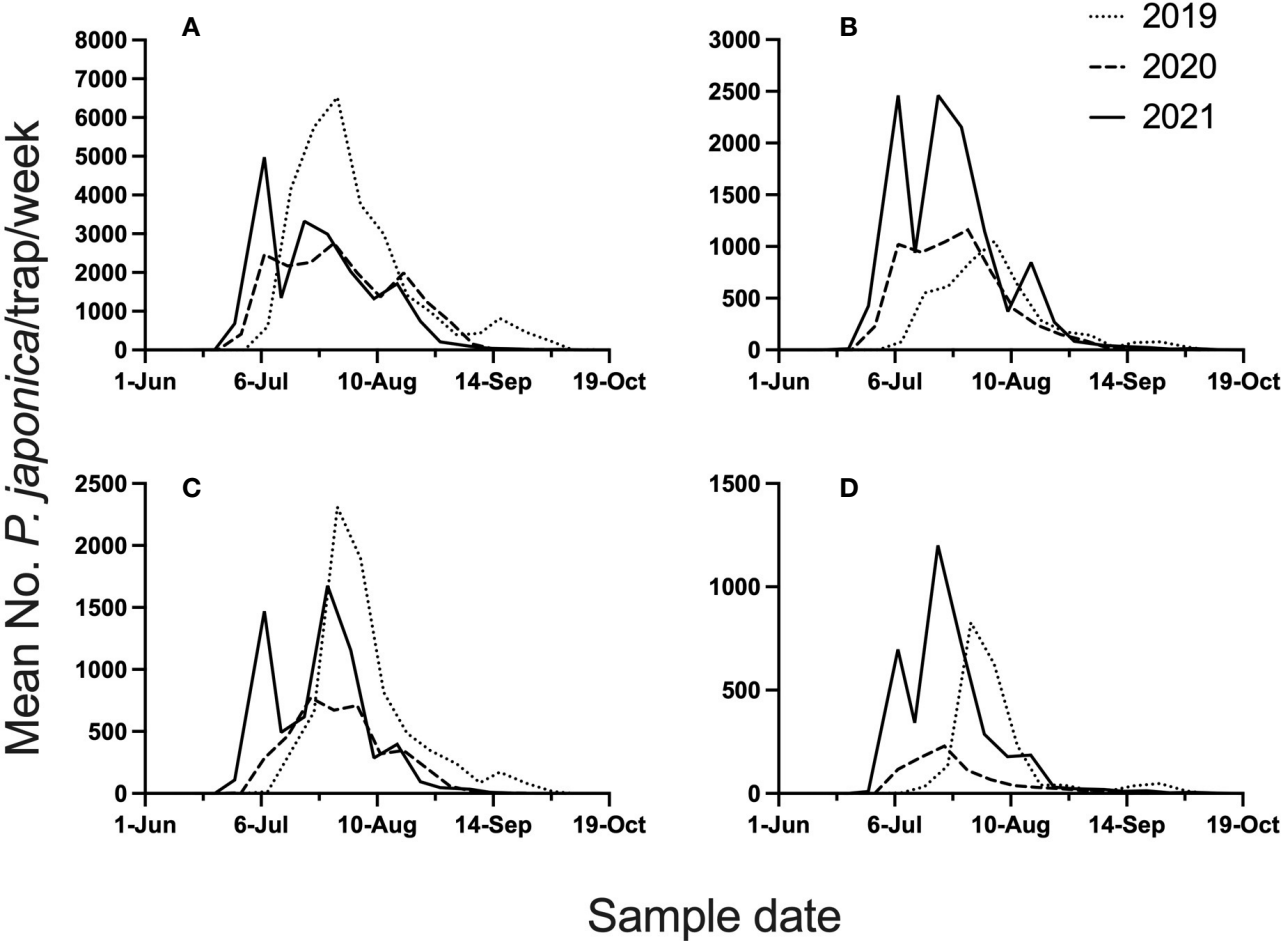
Mean number of *P. japonica* adults per trap, per week, for 2019-2021. Traps were deployed at Rosemount **(A)**, Excelsior **(B)**, Hastings **(C)**, and Forest Lake **(D)**, MN.

The 3-year study provided high *P. japonica* adult populations, with 111,497 beetles caught in semiochemical based traps across all site years. Mean beetle phenology (mean beetle trap catch/week), across the 4 locations and 3 years, is illustrated in **Figure 1**. Given the 3-year study, total beetles captured in datasets for model development were 42,968, whereas validation datasets included 68,571 beetles. Across all years, and for all locations, peak trap-catch on a calendar time scale varied considerably. Mean trap catch was lowest in 2020.

### Model development and validation

**Figure 2:**
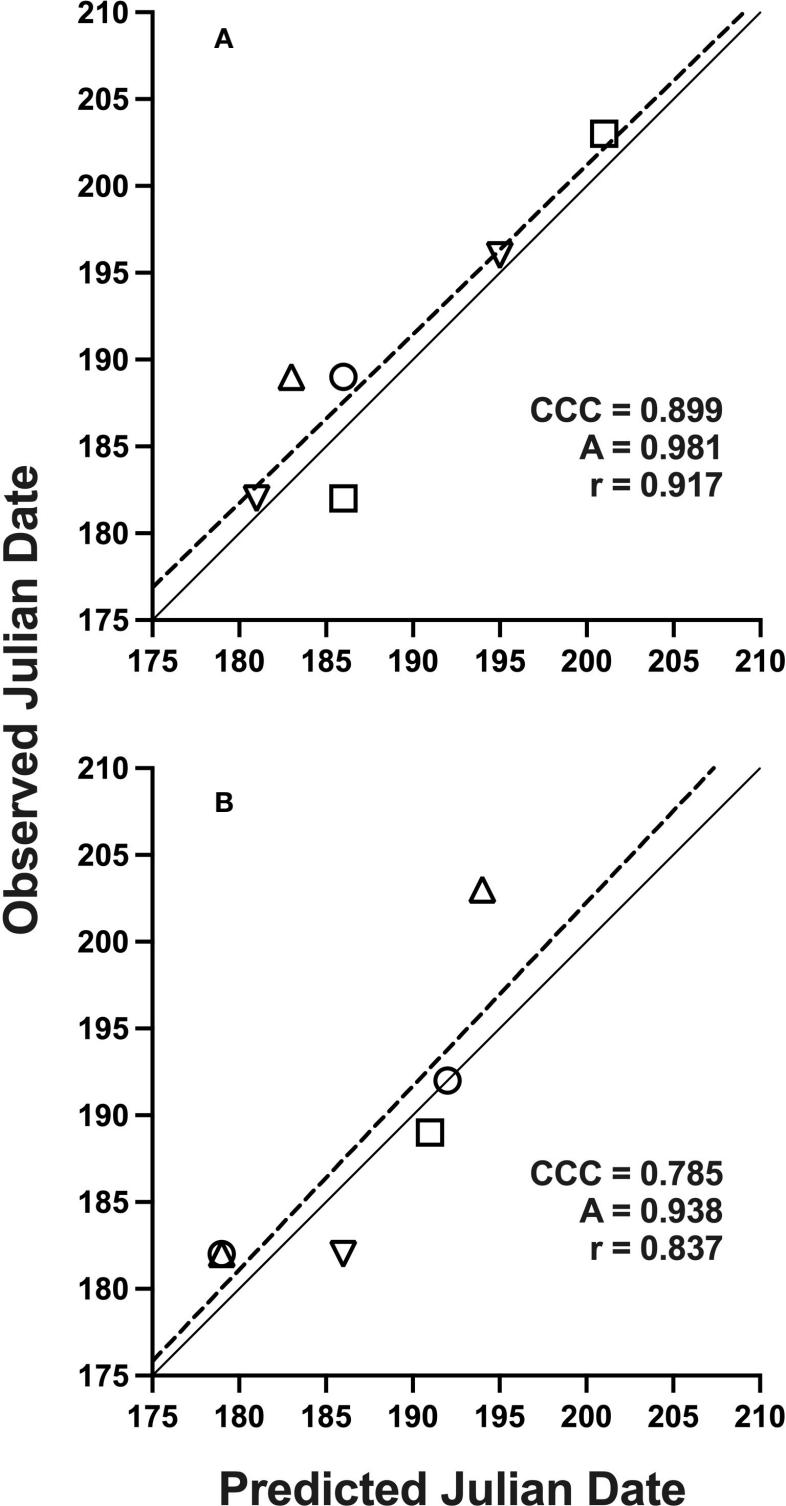
Solid lines indicate perfect agreement. Dashed lines are least squares regression lines that indicate deviations from agreement. Overall agreement is characterized by concordance correlation coefficients (*CCC*), which depend on component measures of precision (Pearson’s correlation coefficient, *r*) and accuracy **(A)**, as defined in text. Data represents Julian date of 10% trap-catch with included *CCC* values using a lower threshold of 15 °C and upper threshold of 21.7 °C for model development **(A)** and validation **(B)**; locations are represented by: (○) Rosemount, (□) Forest Lake, (△) Hastings, (▽) Excelsior, MN, 2019-2021.

Development of the model indicated that the best model for simple average and sine-wave calculation methods performed similarly at 10% emergence (*CCC* = 0.899 and 0.895 respectively), and both had near-perfect agreement based on *CCC* alone (**Table 2**). However, when considering both the highest *CCC* and lowest *AIC* values to measure fit across the entire distribution (**Table 2**), the simple degree-day method, along with recommended lower and upper thresholds, was selected as the most robust model (**Figure 2A**).

**Table 2:** Modeling results to determine the optimum simple *vs*. sine-wave degree-day models, for 4 Minnesota locations, 2019-2021.

Lower threshold (°C)	Upper threshold (°C)	Method	Start date	*AIC*	*CCC*
15.0	21.7	Simple	1	46.226	0.899
15.0	21.7	Simple	32	46.226	0.899
15.0	21.7	Simple	50	46.226	0.899
15.0	21.7	Simple	91	46.226	0.899
15.0	22.2	Simple	1	47.068	0.899
15.0	22.2	Simple	32	47.068	0.899
15.0	22.2	Simple	50	47.068	0.899
15.0	22.2	Simple	91	47.068	0.899
15.0	22.8	Simple	1	47.979	0.899
15.0	22.8	Simple	32	47.979	0.899
15.0	22.8	Simple	50	47.979	0.899
15.0	22.8	Simple	91	47.979	0.899
14.4	23.3	Simple	1	48.599	0.899
14.4	23.3	Simple	32	48.599	0.899
14.4	23.3	Simple	50	48.599	0.899
14.4	23.3	Simple	91	48.599	0.899
11.7	34.4	Sine-wave	50	51.261	0.895
11.7	34.4	Sine-wave	1	51.262	0.895
11.7	34.4	Sine-wave	32	51.262	0.895
11.7	35.0	Sine-wave	50	51.308	0.895

Best performing model was determined by first selecting for the highest CCC, and then lowest AIC values. This table shows the top 20 models out of the 7,392 iterations created.

The best-performing, or optimum model using simple degree-days, was based on lower and upper thresholds of 15 and 21.7 °C, respectively, with a start date of Jan. 1. All start dates used in model development did not exhibit any differences in *CCC*, so the Jan. 1 start date was selected as the biofix date (**Table 2**). Compared to this simple method model (*CCC =* 0.899), multiple sine-wave models did have a similar *CCC* value (*CCC =* 0.895) at 10% emergence. However, *AIC* values (i.e., smaller values indicate better agreement) for these sine-wave models indicated relatively poorer fit across the entire distribution of emergence (**Table 2**). The selected simple method model had an *AIC* of 46.23, which ranked 1,137 out of 7,392 total models for fit across the entire distribution or fell in the top 89% of the range of *AIC* values of 44.46 to 60.61. The top ranked model based on *AIC* alone (*AIC* = 44.46) had a slightly better AIC than the simple model, but also had a poorer *CCC* of 0.745 at 10% emergence. The top-ranked sine-wave model for *CCC* however, had an *AIC* of 51.26 and ranked much lower for 6,195 out of 7,392 models when sorted by *AIC* alone.

Using these selected parameters for simple degree-days, no significant differences were observed when adding a site-year covariate interaction to intercept [*F* (5, 141) = 1.03, *P* = 0.40] or the degree-day effect [*F* (5, 141) = 0.11, *P* = 0.99], which indicated similar model performance of the degree-day effect alone across site-years. When performing logistic regression using only natural-log transformed degree-days as an independent variable, intercept and slope terms were -43.34 and 7.41, respectively [eq. 1]. Additionally, mean differences between predicted and observed days for 10% trap-catch, for model development and validation was limited to -1.4 d (**Table 3**).

**Table 3:** Summary statistics for predicted versus observed days when 10% trap-catch by *P. japonica* occurred among 12 site-years for both model development and validation using the top simple degree-day model.

	Mean error (predicted-observed)	Std. Dev	Min	Max
**Development**	-1.5	3.271085	-6	4
**Validation**	-1.5	4.593474	-9	4

**Figure 3:**
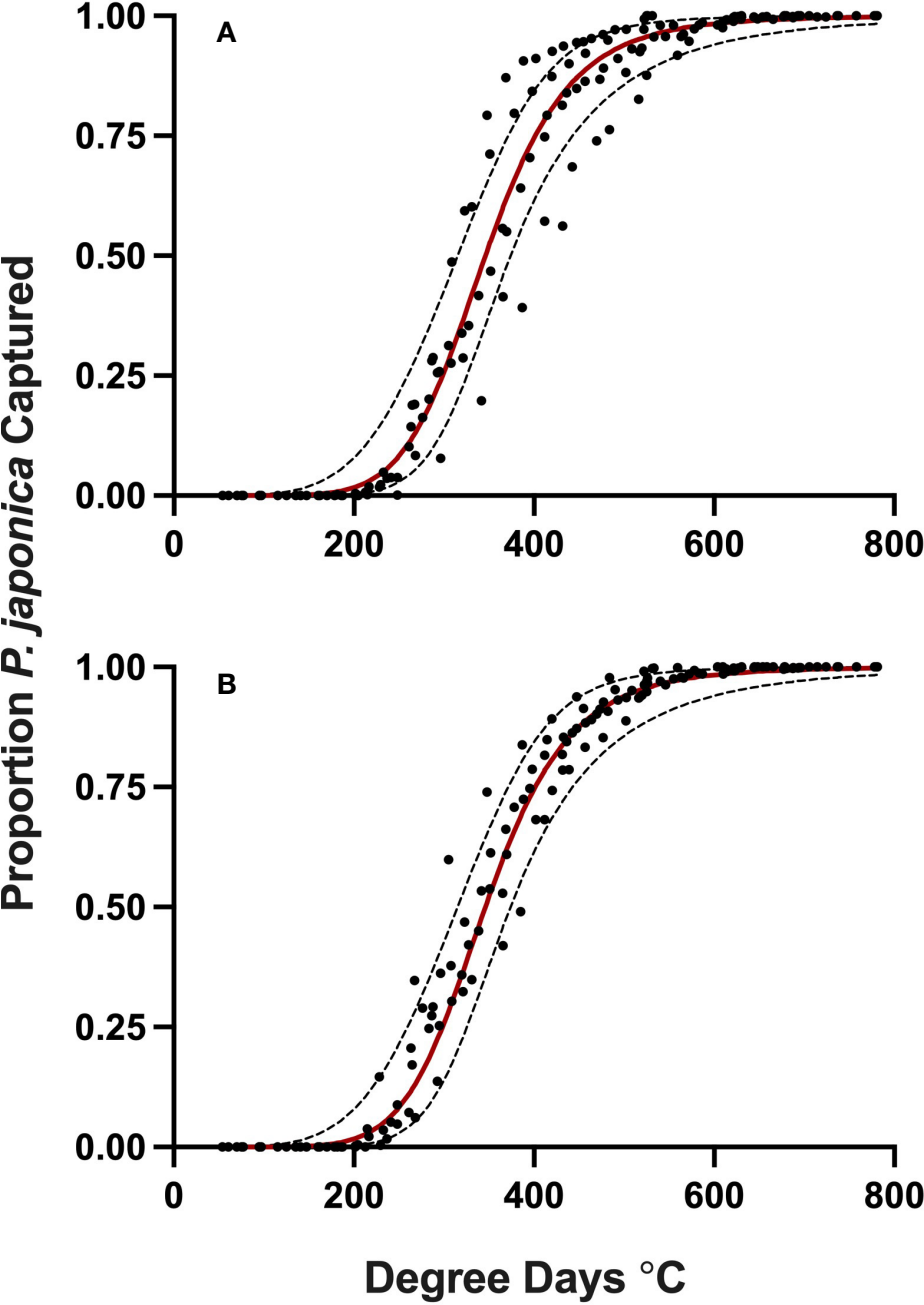
Proportion *P. japonica* adult trap-catch for development **(A)** and validation **(B)** of a degree-day model, in relation to cumulative degree-days, with 95% confidence intervals (red line, predicted model) simple average degree-day model, with a 1 Jan. start date, lower threshold of 15 °C and an upper threshold of 21.7 °C, MN, 2019-2021.

**Figure 4:**
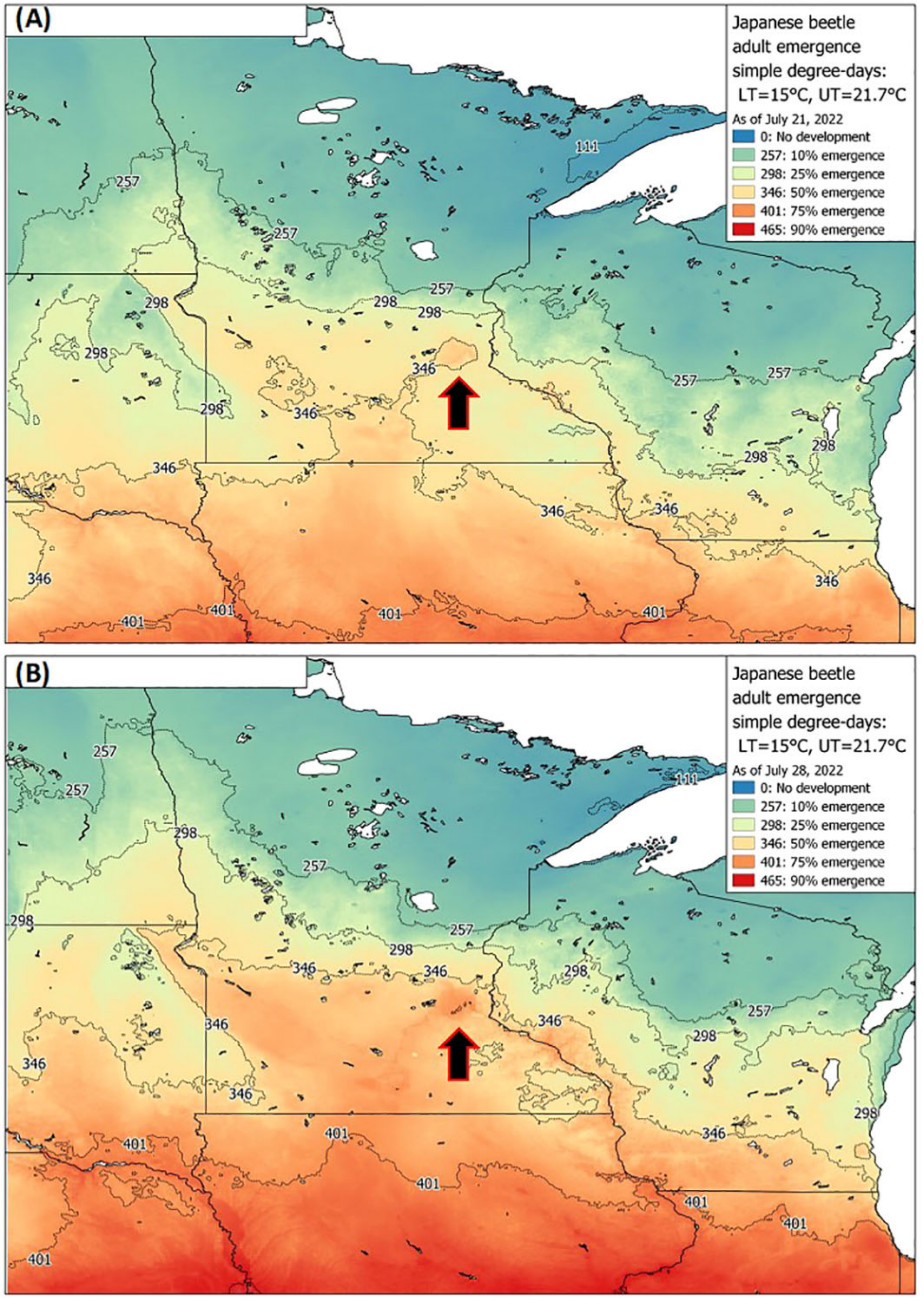
Example of a *P. japonica* predictive tool available to growers and crop consultants, *via* UMN Extension, showing real-time degree-day forecasts for 10-75% emergence as of July 21^st^ **(A)** and July 28^th^ **(B)** based on adult trap-catch, across Minnesota and nearby states during 2022. Maps are based on the simple average degree-day model, with lower and upper thresholds of 15 °C and 21.7 °C, respectively. Arrows indicate the 7-county metro area of Minneapolis-St. Paul, MN, which illustrates earlier beetle trap-catch **(A, B)**, compared to many of the surrounding rural areas.

Datasets used for model validation indicated a lower *CCC* of 0.785 (**Figure 2B**) but were still in strong agreement with an *r* of 0.837 and an *A* of 0.938. When degree-day accumulation to proportion trap-catch data were plotted on the log-logistic distribution, we observe a good fit for both model development and validation data sets (**Figures 3A, B**). Finally, fitting the data to the proposed model indicates that at 257 degree-days (Celsius), adult *P. japonica* populations will have reached 10% trap-catch for the season. Furthermore, this model can be utilized to create spatio-temporal maps adult trap catch phenology, *via* the University of Minnesota VegEdge website (https://vegedge.umn.edu/degree-day-models-select-insect-pests-midwest-region). The software collects 2.5-km^2^ resolution daily temperature data to generate 7-day forecasts, provided through the National Phenology Network (https://www.usanpn.org/data/agdd_maps), and is used to produce pest development maps (**Figure 4**). For the 2022 example shown in **Figure 4A**, the degree-day forecast illustrates an early “hot spot” of adult activity in the 7-county, Minneapolis-St. Paul, metro area, compared to several surrounding rural areas.

## Discussion

Development and validation of a degree-day model for *P. japonica* indicated that a biofix date of 1 Jan., using the simple average degree-day method was the most precise and accurate model at 10% emergence, had improved agreement across the entire emergence curve compared with the best 10% emergence sine-wave model (**Table 2**). Overall, for the simple average model, start dates did not differ in their *CCC* values (**Table 2**); thus, Jan. 1 was selected as a start date to simplify when degree days should begin to be monitored for the season.

In some cases, sine-wave models did have a better fit across the whole emergence distribution than the selected simple model when sorting by *AIC* alone. However, using only this metric sacrificed fit at the target 10% emergence needed to alert growers in a timely fashion. Instead, prioritizing fit at 10% emergence by first sorting by *CCC* resulted in a simple model providing the best prediction out of all models at 10% emergence while still providing good prediction across the entire distribution where the selected model ranked 1,137 out of 7,392 total models for *AIC* (**Table 2**; **Figure 3**). *AIC* values do not indicate absolute measures of agreement like *CCC*, but are useful for making relative comparisons between models. These results also help illustrate the need to use multiple measures of agreement in modeling, especially when needing to optimize targeting a specific point on the emergence curve for IPM planning versus modeling the whole emergence period for wider uses. In addition to increased overall model performance in this instance, the simple degree-day calculations are less complex than sine-wave, so the simple degree-day model is likely to have a higher chance of adoption for use by growers (34). We therefore recommend beginning the simple average degree-day accumulation on Jan. 1 using a lower threshold of 15°C (59 °F) and upper threshold of 21.7 °C (71 °F).

This model will be useful in predicting 10% beetle trap-catch (257 degree-days, C), which should be early enough in the season to give growers and crop consultants ample time to prepare for in-field sampling, and advance warning of peak beetle activity. The model can also forecast a reasonable estimate of beetle trap-catch at 25%, 50% (peak), 75% and 90%, for degree days of 298, 346, 401, and 465 (°C), respectively (**Table 4**). Knowledge of these degree-day forecasts will be useful for estimating the onset and phenology of trap-catch within a given year, and for comparing phenology across years.

**Table 4:** Degree-day estimates for the simple model, in °C and °F in relation to predicted adult trap-catch of P. japonica*.

Proportion trap-catch	Degree-days °C	Degree-days °F
0.10	257	463
0.25	298	537
0.50	346	623
0.75	401	722
0.90	465	837

*Simple degree-day model using lower and upper thresholds of 15 and 21.7C, respectively (see **Table 2**). Proportion catch can be calculated using eq. 1 with °C degree-days, intercept -43.34 and slope 7.41. Degree-day requirements, from C to F, where F = C * 9/5 (19, 34).

To date, only a few previous studies have used laboratory-based developmental rates, and modeling to predict development of immature stages, to estimate *P. japonica* adult emergence or phenology (12, 35, 36). While these studies provided new insights into specific developmental thresholds for *P. japonica* life stages, it is difficult to compare the previous results with our field-based results. Our study, conducted in 2019-2021, used only ambient air temperatures collected from nearby weather stations and trap-catch data in the modeling analysis to attempt to characterize development of individuals in the soil. Studies conducted by Régniére et al. (12) placed larvae in individual cups and used laboratory-controlled temperatures to record the immature development of *P. japonica.* Further studies in the laboratory were conducted to attempt to assess adult maturation and time to emergence from rearing medium for several constant temperatures (12). While this information is important to understanding the biology of larvae, and adult eclosion, our research aimed to assist with predicted timely beetle emergence and phenology for IPM applications under field conditions, often involving multiple unknown variables.

In our study, we initiated data collection after *P. japonica* adults eclosed and found that this approach could be useful in forecasting the adult life stage and are meant to be used as a tool for monitoring and tracking the adult population, versus previous studies which looked to measure development. Our study was designed to allow potential confounding factors from field conditions that can act as nuisance variables to be incorporated as background variation across the selected variables: calculation method, upper and lower threshold, and biofix date. This allowed us to use air temperature, a more accessible type of weather data than soil temperature and determine if air temperature alone could reliably predict development while immature stages develop in the soil. However, the tradeoff is that is our model parameters, such as lower thresholds, are purposely confounded either with environmental effects, such as differences in soil and air temperatures or soil moisture, or as interactions with the other model terms. While this allows development of models with simple inputs to represent complex field conditions, our parameters will not necessarily be directly comparable to previous studies in controlled laboratory conditions.

Understanding the differences in the aim of each study helps to better understand why lower and upper thresholds appear to be so different across the studies. Ambient air and soil temperatures are closely related, but depending on the height both above and below the soil surface where measurements are taken, there can be substantial differences observed between air temperature and what the insect actually experiences. These differences, particularly in temperate regions, often occur during spring, when soils warm relatively slowly compared to ambient air temperatures (19). Also, air temperatures can change drastically depending on cloud cover, precipitation events and changes in solar radiation (37). By contrast, each of these factors demonstrate what soil-dwelling insects are going to experience in different developmental environments when compared to adult life stages that have completed development and emerged from the soil (38). Similar to traditional regression analyses, background variation in an iterative modeling study such as this can cause parameters, such as lower thresholds based on air or soil temperatures, to deviate from thresholds determined in controlled laboratory studies (e.g. 19). For instance, the best sine-wave model did have a lower threshold of 11.7°C, which is closer to the lower threshold of 10°C described by Régnière et al. (12) than the simple model’s lower threshold of 15°C. The thresholds in our models may be different than laboratory studies due to the buffering effects of the soil, differences in how simple and sine-wave calculations accumulate degree-days, the interaction of those two effects (e.g., how well each method accounts for soil buffer effects), or any number of other field or model parameter combinations. Because each model’s performance is dependent on the combination of calculation method, upper and lower threshold, and to a lesser extent for the top models in **Table 2**, start date, the effect of a single parameter cannot be easily compared in isolation to laboratory study thresholds. Other approaches could include measuring soil temperature directly in a study such as this. However, ambient air temperature measurements are much more accessible relative to observed or modeled soil temperatures, which makes it easier for growers and producers to efficiently monitor their own degree-days.

Additionally, understanding the behavior of adult *P. japonica* after emergence can help explain why in **Figures 3A, B**, we observed a slight increase in the spread of observed data points as the model approaches 50% trap-catch. We speculate that this occurs for at least two reasons. At 10% trap-catch, or soon after adult eclosion and emergence, male beetles have specific tendencies to find a newly emerged, virgin female for mating (12), and will soon be attracted to the pheromone baited traps, following mating. These behaviors may explain why we see such a tight fit to the predicted model as both males and females will emerge from the soil and stay close to their emergence site for mating and initial flights to traps. Once mating has occurred and virgin females are no longer the majority, males will begin to expand their behavior to prioritize feeding on suitable hosts, and potentially move significant distances to seek preferred host plants such as wild grapes, wine grapes, or raspberry (9, 39–41). The variation observed in data point spread is an indication of this phenomenon as trap catch may now be accounting for beetles that have immigrated from other emergence sites to find suitable hosts for feeding and mating. Furthermore, progressing through the season, beetle behavior and flight is heavily dependent on other environmental factors such as cloud cover, wind, rainfall, and humidity (9, 42). While these environmental factors are still important after first emergence, beetles may not be traveling to other host sites as much due to their priority of mating with virgin females in the immediate area. However, even with the wider distribution of data points as degree-days reach the 50% activity, the model is still a beneficial predictive tool, especially considering how indiscriminate beetle behavior and dispersal becomes after initial mating goals have been met. In addition, the increase in variation is occurring after the primary target of predicting 10% trap-catch, as an early warning forecast prior to peak beetle trap-catch at 50%.

Efficient monitoring of crop pests is fundamental to the success of IPM programs, particularly for invasive arthropod species (43). Development of this degree-day model for *P. japonica* was created with the objective to produce an additional tool to assist growers, crop consultants, and regulatory staff, with an early-warning and predictive method for tracking beetle population phenology more efficiently. Calculations using a simple degree-day method, with the recommended lower (15 °C) and upper (21.7°C) thresholds, and a biofix date of 1 Jan. will provide growers and producers an early warning forecast for when 10% trap-catch will occur, and when crops should be monitored more closely for potential feeding damage (e.g., 17). Even with potential background sources of environmental variation, this model had very high agreement at the target 10% emergence predictions, and was one of the better performing models across the entire emergence period, which indicates this model can be a reliable and tool with relatively simple inputs for growers.

As the accumulation of degree-days reaches 257 °C, growers and regulatory staff can also prepare for the predicted peak beetle trap-catch, at 346 °C. The recent example, *via* the University of Minnesota Extension, VegEdge website (https://vegedge.umn.edu/degree-day-models-select-insect-pests-midwest-region), is the regional map of *P. japonica* beetle phenology, illustrated in **Figure 4**. The regional map is updated daily during the growing season using the simple average degree-day model presented in **Figure 4**. The degree-day maps provided real-time updates during the growing season for both 10% and 50% trap-catch targets; in addition, 7-day forecasts are also provided (e.g., www.fruitedge.umn.edu), to assist growers in tracking *P. japonica* adult activity. Although our model will be of immediate use in Minnesota, and likely perform well in other temperate climates, future applications beyond the region may require additional validation. Additional research could also be directed toward improved understanding of the development and survival of the larval instars during spring in relation to warming soil temperatures, and for a variety of climate regions. This could assist with further understanding of the efficacy of various management strategies against the larval stage, the role of overwintering soil temperature stress the larval stage (44), as well as improvements in phenology models, particularly in newly invaded regions or countries. Finally, the degree-day model presented here should continue to be useful to growers for *P. japonica* management in the future, given the context of global climate change for the Midwest region. For example, in Minnesota, ambient temperatures over next 80 years are projected to increase by 4 to 6 °C, during summer and winter periods, respectively (22).

## Data availability statement

The raw data supporting the conclusions of this article will be made available by the authors, without undue reservation.

## Author contributions

DE, AH and WH: conceptualization. WH and EB: resources, project administration, and funding acquisition. DE, AH, EB: methodology. DE and AH: investigation and formal analysis. DE and AH: writing original draft, and preparation. EB, and WH: writing and reviewing. All authors contributed to the article and approved the submitted version.
